# Relationships Between Changes in Serum Ketone Body Levels and Metabolic Effects in Patients with Severe Obesity Who Underwent Laparoscopic Sleeve Gastrectomy

**DOI:** 10.1007/s11695-024-07337-8

**Published:** 2024-06-06

**Authors:** Akira Umemura, Akira Sasaki, Hideki Kumagai, Yota Tanahashi, Takafumi Iwasaki, Hiroyuki Nitta

**Affiliations:** Department of Surgery, Iwate Medical University School of Medicine, 2-1-1 Idaidori, Yahaba, Iwate 028-3695 Japan

**Keywords:** Laparoscopic sleeve gastrectomy, Ketone body, Type 2 diabetes, Metabolic dysfunction–associated steatohepatitis, Chronic kidney disease

## Abstract

**Background:**

Serum ketone bodies increase due to dynamic changes in the lipid metabolisms of patients undergoing bariatric surgery. However, there have been few studies on the role of ketone bodies after bariatric surgery. We aimed to clarify the role of and relationship between the changes in serum ketone bodies and weight loss, as well as between those changes and the metabolic effects after laparoscopic sleeve gastrectomy (LSG).

**Methods:**

We recruited 52 patients with severe obesity who underwent LSG. We measured acetoacetic acid (AcAc) and β-hydroxybutyric acid (β-OHB) at the baseline, 1 month, and 6 months after LSG. Subsequently, we compared the changes in the serum ketone bodies with weight-loss effects and various metabolic parameters.

**Results:**

At 1 month after LSG, β-OHB significantly increased (*p* = 0.009), then significantly decreased 6 months after LSG (*p* = 0.002). In addition, β-OHB in patients without Type 2 diabetes (T2D) and metabolic dysfunction-associated steatohepatitis (MASH) was notably higher than in patients with T2D at 1 month after LSG (*p* < 0.001).

In the early phase, both AcAc and β-OHB mainly had strong positive correlations with changes in T2D- and MASH-related parameters. In the middle term after LSG, changes in both AcAc and β-OHB were positively correlated with changes in lipid parameters and chronic kidney disease-related parameters.

**Conclusion:**

We demonstrated that the postoperative surge of ketone bodies plays a crucial function in controlling metabolic effects after LSG. These findings suggest the cause- and consequence-related roles of ketone bodies in the metabolic benefits of bariatric surgery.

**Graphical Abstract:**

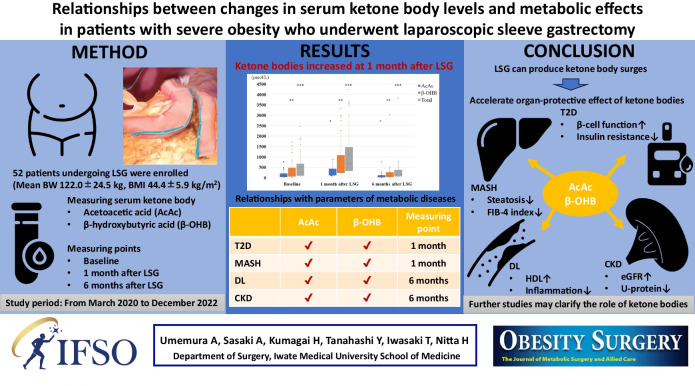

## Introduction

Bariatric surgery produces various effects on intercellular signaling pathways via prompt changes in metabolic parameters just after surgery. These changes, for example, the incretin effect, the reduction of lipotoxicity, the improvement of gut microbiota, and the reduction of oxidative stress can be explained and have been demonstrated in previous articles [1–4]. However, none of these can produce a metabolic effect alone but must combine to result in strong weight loss and mitigate metabolic diseases.

Ketone bodies are endogenous metabolites mainly produced by the liver, particularly under conditions of prolonged fasting, insulin deprivation, and extreme exercise [5]. The conditions just before bariatric surgery and the early postoperative phase in patients with severe obesity are also similar; therefore, serum ketone bodies notably heighten in patients undergoing bariatric surgery [6]. Recent studies have demonstrated that more serum ketone bodies may be beneficial for cardiovascular disease and have renoprotective functions [7–9]. Against this background, changes in serum ketone bodies after bariatric surgery may play a key role in producing metabolic effects.

In this study, we aimed to clarify the relationship between changes in serum ketone body levels and weight loss/metabolic effects in patients with severe obesity who underwent laparoscopic sleeve gastrectomy (LSG).

## Patients and Methods

This research was designed as a single-center prospective cohort study. Herein, 52 patients with severe obesity who underwent LSG were enrolled in this study from March 2020 to December 2022. All patients met the following inclusion criteria for metabolic and bariatric surgery treatment established by national health insurance practice: between 18 and 65 years of age, severe obesity (body mass index [BMI] > 35 kg/m^2^), and the presence of at least one obesity-related disease with resistance to medical treatment (hypertension, type 2 diabetes [T2D], dyslipidemia, and obstructive sleep apnea). Exclusion criteria were history of alcohol abuse, secondary obesity (drug-induced or due to endocrine diseases), and the presence of uncontrolled psychiatric disorders.

Regarding surgical procedures, the LSGs involved 70–80% gastric-volume reduction by resecting the stomach alongside a 36-Fr bougie, beginning 4 cm from the pylorus and ending at the angle of His. All patients were continuously evaluated and cared for by a multidisciplinary team from the initial visit to postoperative follow-up to improve weight loss and metabolic effects. In this study, patients underwent LSG were enrolled for equalizing weight-loss and metabolic effects.

The study protocol followed the guidelines of the 1975 Declaration of Helsinki and was approved by the Institutional Ethics Committee (H27-42, MH2022-056). We obtained written informed consent from all participants at enrollment.

A prospective database was used to store the following data from all subjects at their baseline, 1 and 6 months after LSG: body weight, BMI, total weight loss (TWL), subcutaneous fat area (SFA), visceral fat area (VFA), and liver volume (LV) to evaluate weight-loss effects; fasting glucose, immunoreactive insulin, hemoglobin A1c (HbA1c), a homeostasis model of assessing insulin resistance, a homeostatic model assessment of beta cell function, and C-peptide to evaluate T2D; aspartate aminotransferase (AST), alanine aminotransferase, ferritin, Type IV collagen 7S, transferrin, and FIB-4 index to evaluate metabolic dysfunction-associated steatotic liver disease (MASLD) [10]; total cholesterol (TC), triglyceride (TG), low-density lipoprotein cholesterol (LDL-C), high-density lipoprotein cholesterol (HDL-C), and high-sensitivity C-reactive protein (HS-CRP) to evaluate dyslipidemia; and estimated glomerular filtration rate (eGFR), blood urea nitrogen (BUN), creatinine, serum aldosterone, urinary protein (U-protein), and urinary albumin (U-albumin) to evaluate chronic kidney disease (CKD). In this study, we examined U-protein and U-albumin using first-spot urine in the morning. We measured serum acetoacetic acid (AcAc) and β-hydroxybutyric acid (β-OHB) using the enzyme cycling method, and we presented the total ketone body by adding these two parameters.

Regarding parameters requiring image analysis, SFA, VFA, and LV were calculated through computed tomography (CT) when laboratory data were collected. These calculations used a 64-row CT (AquilionTM; Toshiba Medical Systems Corporation, Tokyo, Japan). After obtaining a single tomographic slice at the umbilicus level, VFA and SFA were detected and measured using a Hounsfield unit (HU) range for adipose tissue of − 150–0 HU and were then calculated and recorded in cm^2^. To measure LV, CT images were downloaded as digital images and copied from medical files to a computer workstation (SYNAPSE VINCENT; Fujifilm; Tokyo, Japan). The LV was recorded in milliliters.

To evaluate the histopathological findings of MASLD, intraoperative liver biopsies were performed on all participants. In principle, an ultrasound-guided liver biopsy for patients with severe obesity is risky due to the patients’ thick subcutaneous fat tissue; therefore, a preoperative ultrasound-guided liver biopsy is usually avoided, except for special reasons. The biopsy specimens were formalin-fixed and stained with hematoxylin–eosin, silver reticulin, and Masson trichrome to check for liver fibrosis. The histopathological findings were recorded using steatosis percentage, NAFLD activity score, and pericellular fibrosis score [11, 12]. These histopathological findings were evaluated by a specialized pathologist, and the diagnosis of metabolic dysfunction-associated steatohepatitis (MASH) was determined using the fatty liver inhibition of the progression algorithm [13].

## Statistical Analysis

This study’s data were recorded as numbers, percentages for categorical variables, and means ± standard deviations for continuous variables. Statistical analyses were performed using Student’s t-tests or Mann–Whitney U tests for continuous variables. The correlations between ketone bodies and weight loss, as well as the metabolic effects, were analyzed using Spearman’s correlation coefficient. We considered *p*-values of less than 0.05 statistically significant, and all reported *p*-values were two-sided. All statistical analyses were performed using JMP Pro Version 15 (SAS Institute Inc., Cary, NC, USA).

## Results

The patients’ characteristics at baseline and the histopathological prevalence of MASH are presented in Table 1. The enrolled patients’ mean initial body weight and BMI were 122.0 kg and 44.4 kg/m^2^, respectively. Concerning obesity-related diseases, 25 and 41 of the enrolled patients had T2D and histopathological MASH, respectively, according to intraoperative liver biopsies.

**Table 1:** Clinical characteristics of enrolled patients

Variables	
Age (years)	38.5 ± 10.3
Gender (Male / Female)	23/29
Initial body weight (kg)	122.0 ± 24.5
Initial BMI (kg/m^2^)	44.4 ± 5.9
Obesity-related disease, n (%)	
T2D	25 (48.1)
Histopathological MASH	41 (78.8)
Hypertension	38 (69.2)
Dyslipidemia	40 (76.9)
Hyperuricemia	33 (63.5)
Obstructive sleep apnea	50 (96.1)

*BMI*, body mass index; *T2D*, type 2 diabetes; *MASH*, metabolic dysfunction-associated steatohepatitis

Table 2 also presents the weight loss and metabolic effects at every measurement point. The mean body weight loss and BMI loss 6 months after LSG were 30.9 kg (*p* < 0.001) and 11.2 kg/m^2^ (*p* < 0.001), respectively. Meanwhile, the mean TWL was 25.0%. Concerning their fat tissue volume, their SFAs, VFAs, and LVs notably decreased (*p* < 0.001). All parameters of T2D significantly improved at 6 months after LSG. Almost all the parameters of MASLD and dyslipidemia also improved except for the FIB-4 index (*p* = 0.406) and TC (*p* = 0.428). In contrast, neither parameter of CKD markedly improved.

**Table 2:** Weight loss effects and improvement of metabolic parameters

Variables	Baseline	1 months after LSG	6 months after LSG	*p*-value
Body weight (kg)	122.0 ± 24.5	101.5 ± 17.7	91.1 ± 17.8	< 0.001
BMI (kg/m^2^)	44.4 ± 5.9	37.0 ± 4.4	33.2 ± 4.5	< 0.001
TWL (%)	-	15.5 ± 5.3	25.0 ± 7.2	-
SFA (cm^2^)	517.2 ± 140.0	409.3 ± 135.2	335.7 ± 108.5	< 0.001
VFA (cm^2^)	240.6 ± 62.6	178.6 ± 58.7	138.1 ± 48.0	< 0.001
LV (mL)	2218.9 ± 402.6	1718.1 ± 296.6	1799.2 ± 294.5	< 0.001
FG (mg/dL)	104.0 ± 29.9	91.4 ± 20.7	89.7 ± 15.4	< 0.001
IRI (μIU/mL)	25.3 ± 23.7	10.9 ± 5.4	9.8 ± 5.0	< 0.001
HbA1c (%)	6.4 ± 1.0	5.6 ± 0.4	5.5 ± 0.5	< 0.001
HOMA-IR (no unit)	6.5 ± 7.9	2.4 ± 1.3	1.8 ± 1.4	< 0.001
HOMA-β (no unit)	223.2 ± 229.7	163.5 ± 136.8	137.7 ± 120.1	< 0.001
C-peptide (ng/ml)	3.5 ± 1.9	2.3 ± 1.2	10.0 ± 48.7	< 0.001
AST (U/L)	45.2 ± 35.1	28.2 ± 14.8	19 ± 9.3	< 0.001
ALT (U/L)	58.6 ± 36.7	35.6 ± 24.0	20.2 ± 17.9	< 0.001
Ferritin (ng/mL)	218.7 ± 194.8	214.3 ± 166.0	124.6 ± 124.5	0.008
Type IV collagen 7S (ng/mL)	4.1 ± 1.1	3.9 ± 1.0	3.8 ± 0.8	0.031
Transferrin (ng/mL)	276.8 ± 41.8	222.1 ± 41.7	257.7 ± 51.0	< 0.001
FIB-4 index (no unit)	0.97 ± 1.1	0.84 ± 0.43	0.69 ± 0.32	0.406
TC (mg/dL)	180.1 ± 37.9	155.5 ± 33.4	175.8 ± 34.0	0.428
TG (mg/dL)	133.9 ± 82.4	104.0 ± 36.8	102.2 ± 39.5	0.006
LDL-C (mg/dL)	120.1 ± 31.4	96.1 ± 30.5	106.1 ± 32.2	< 0.001
HDL-C (mg/dL)	40.0 ± 7.7	37.3 ± 6.0	49.1 ± 9.0	< 0.001
HS-CRP (mg/dL)	951.9 ± 1709.7	396.8 ± 443.4	320.6 ± 592.2	0.039
eGFR (mL/min/1.73m^2^)	79.9 ± 11.5	80.3 ± 12.8	80.7 ± 11.0	0.859
BUN (mg/dL)	13.5 ± 3.8	11.0 ± 4.7	12.5 ± 3.5	0.091
Creatinine (mg/dL)	0.73 ± 0.16	0.73 ± 0.2	0.72 ± 0.2	0.429
Aldosterone (pg/mL)	89.8 ± 60.9	90.1 ± 93.6	51.8 ± 47.5	0.083
U-Protein (mg/day)	23.3 ± 75.3	20.5 ± 69.4	19.8 ± 53.0	0.213
U-Albumin (mg/L)	11.6 ± 53.3	10.2 ± 50.8	7.5 ± 28.4	0.211

*LSG*, laparoscopic sleeve gastrectomy; *BMI*, body mass index; *TWL*, total weight loss; *SFA*, subcutaneous fat area; *VFA*, visceral fat area; *LV*, liver volume; *FG*, fasting glucose; *IRI*, immunoreactive insulin; *HbA1c*, hemoglobin A1c; *HOMA-IR*, homeostasis model assessment-insulin resistance; *HOMA-β*, homeostatic model assessment beta cell function; *AST*, aspartate aminotransferase; *ALT*, alanine aminotransferase; *TC*, total cholesterol; *TG*, triglyceride; *LDL-C*, low-density lipoprotein cholesterol; *HDL-C*, high-density lipoprotein cholesterol; *HS-CRP*, high-sensitivity C-reactive protein; *eGFR*, estimated glomerular filtration rate; *BUN*, blood urea nitrogen; *U-protein*, urinary protein; *U-albumin*, urinary albumin

**Fig. 1:**
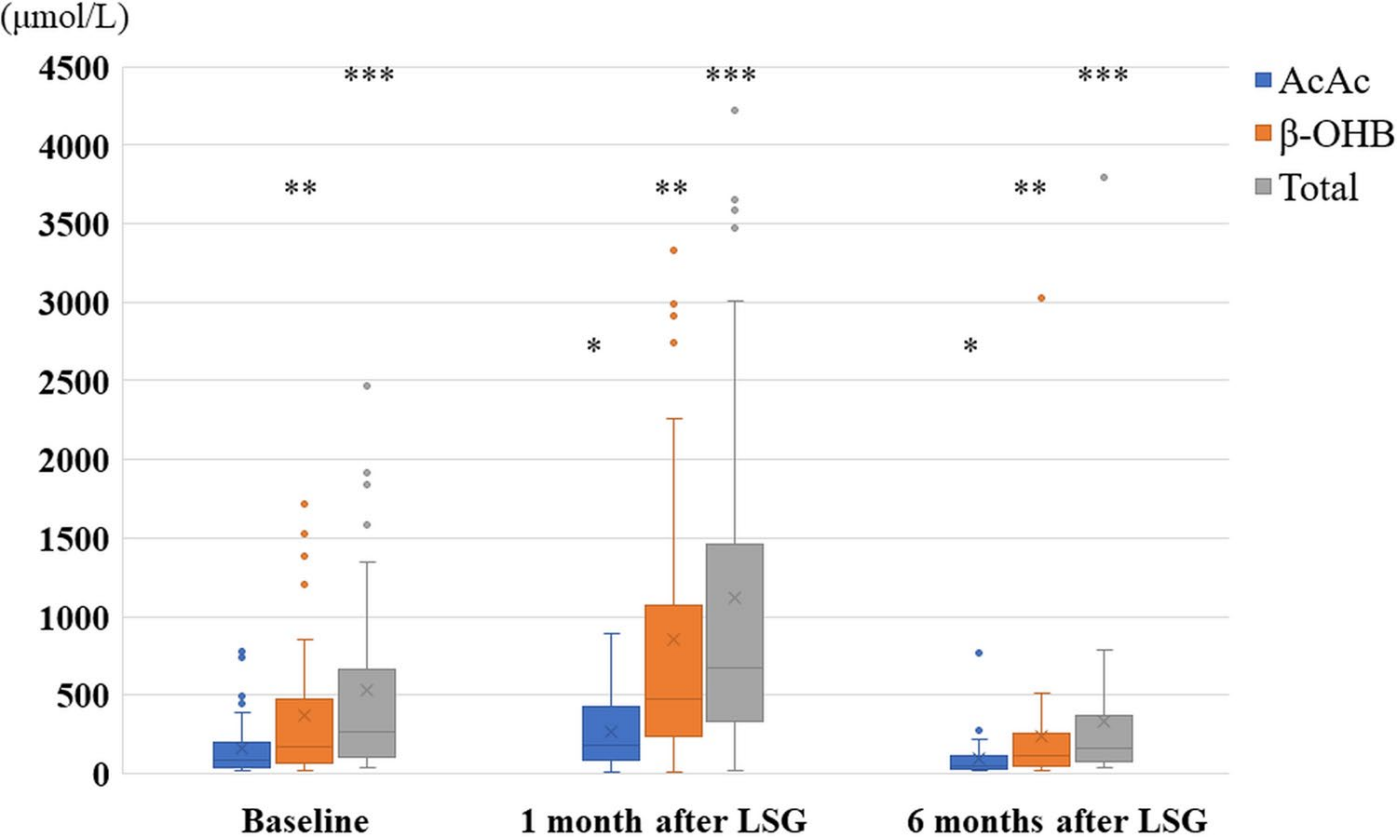
Longitudinal changes of serum ketone bodies in enrolled patients. Each ketone parameter similarly changed at 1 month and 6 months after LSG, showing lower values than the baseline data. * The mean AcAc at 6 months after LSG significantly decreased compared to 1 month after LSG (159.4 μmol/L vs. 270.0 μmol/L, *p* < 0.001). ** The mean β-OHB at 1 month after LSG significantly increased compared to baseline (852.3 μmol/L vs. 369.2 μmol/L, *p* = 0.009), after which it decreased 6 months after LSG (233.7 μmol/L vs. 852.3 μmol/L, *p* = 0.002). *** Total ketone bodies also significantly increased at 1 month after LSG (1122.3 μmol/L vs. 528.6 μmol/L, *p* = 0.018), then decreased at 6 months after LSG (327.5 μmol/L vs. 1122.3 μmol/L,* p* = 0.001)

**Fig. 2:**
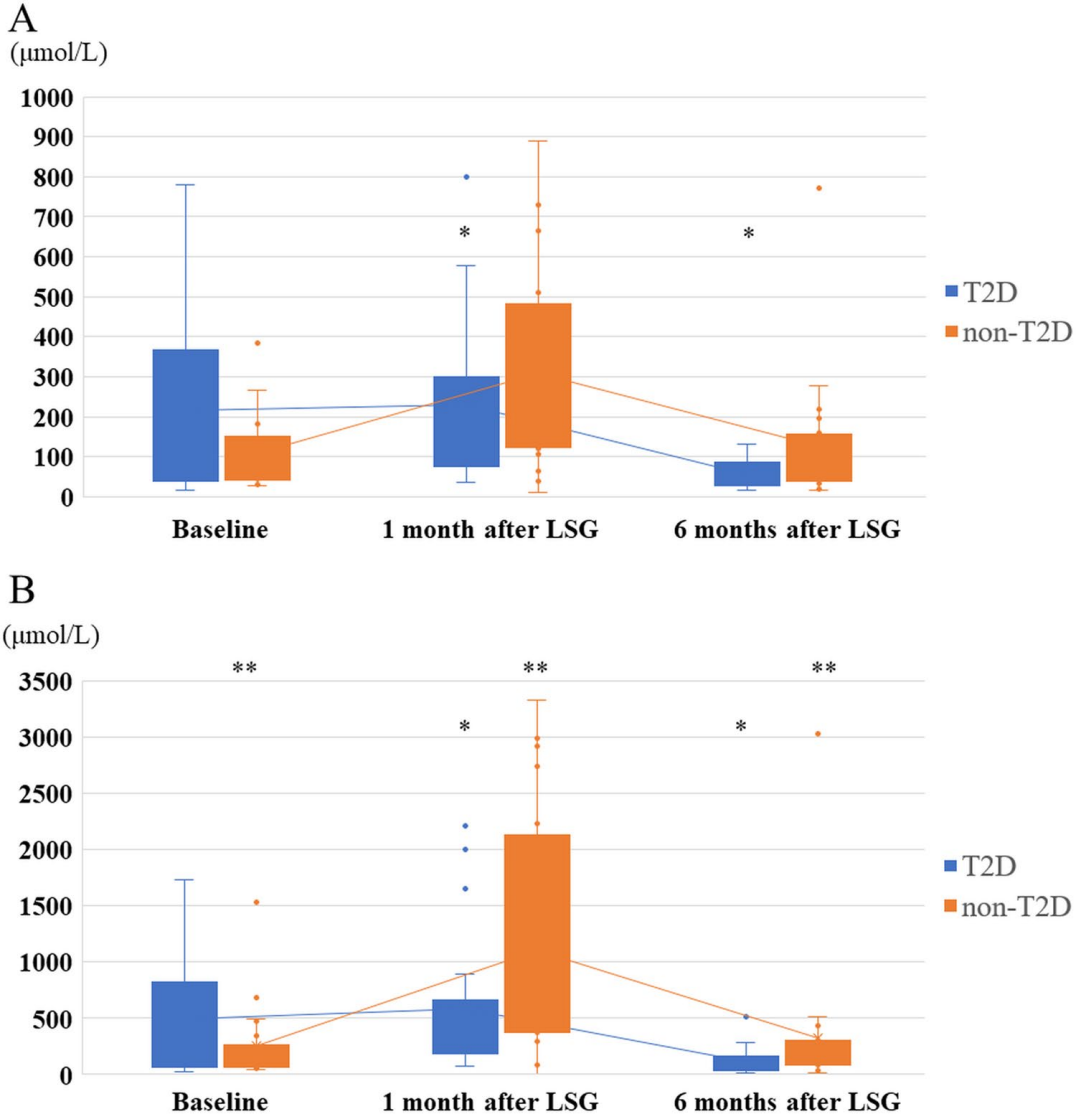
Comparison of AcAc and β-OHB levels between patients with and without T2D. Both AcAc and β-OHB at 1 month after LSG dramatically decreased to a fraction of the baseline in patients with T2D and increased at 1 month after LSG in patients without T2D, then they almost reached the basal value at 6 months after LSG. **A**: Changes in AcAc levels. * Serum AcAc levels in patients with T2D significantly lessened 6 months after LSG (54.5 μmol/L vs. 299.9 μmol/L, *p* = 0.003). **B**: Changes in β-OHB levels. * Serum β-OHB levels in patients with T2D at 6 months after LSG significantly decreased compared to 1 month after LSG (119.1 μmol/L vs. 583.6 μmol/L, *p* = 0.006). ** Serum β-OHB levels in patients without T2D significantly increased at 1 month after LSG (1098.5 μmol/L vs. 258.4 μmol/L,* p* = 0.009), and then significantly decreased at 6 months after LSG (325.4 μmol/L vs. 1098.5 μmol/L, *p* = 0.019)

Figure 1 presents changes in ketone bodies during the research period. Each ketone parameters similarly increased at 1 month after LSG, but also similarly decreased significantly at 6 months after LSG showing lower values than the baseline data. At 1 month after LSG, AcAc increased compared to the baseline; however, it was not significant. In contrast, β-OHB noticeably heightened 1 month after LSG (852.3 μmol/L vs. 369.2 μmol/L, *p* = 0.009), after which it decreased 6 months after LSG (233.7 μmol/L vs. 852.3 μmol/L, *p* = 0.002). The total ketone body changed, the same as β-OHB. Figure 2 presents the changes in ketone bodies in patients with/without T2D. Both AcAc and β-OHB at 1 month after LSG decreased to a fraction of the baseline in patients with T2D, and increased at 1 month after LSG in patients without T2D. On the other hand, they almost reached the basal value at 6 months after LSG. Serum AcAc levels in patients with T2D significantly lessened 6 months after LSG (54.5 μmol/L vs. 299.9 μmol/L, *p* = 0.003). Conversely, β-OHB in patients without T2D was much higher than in patients with T2D 1 month after LSG (1098.5 μmol/L vs. 583.6 μmol/L, *p* < 0.001).

**Fig. 3:**
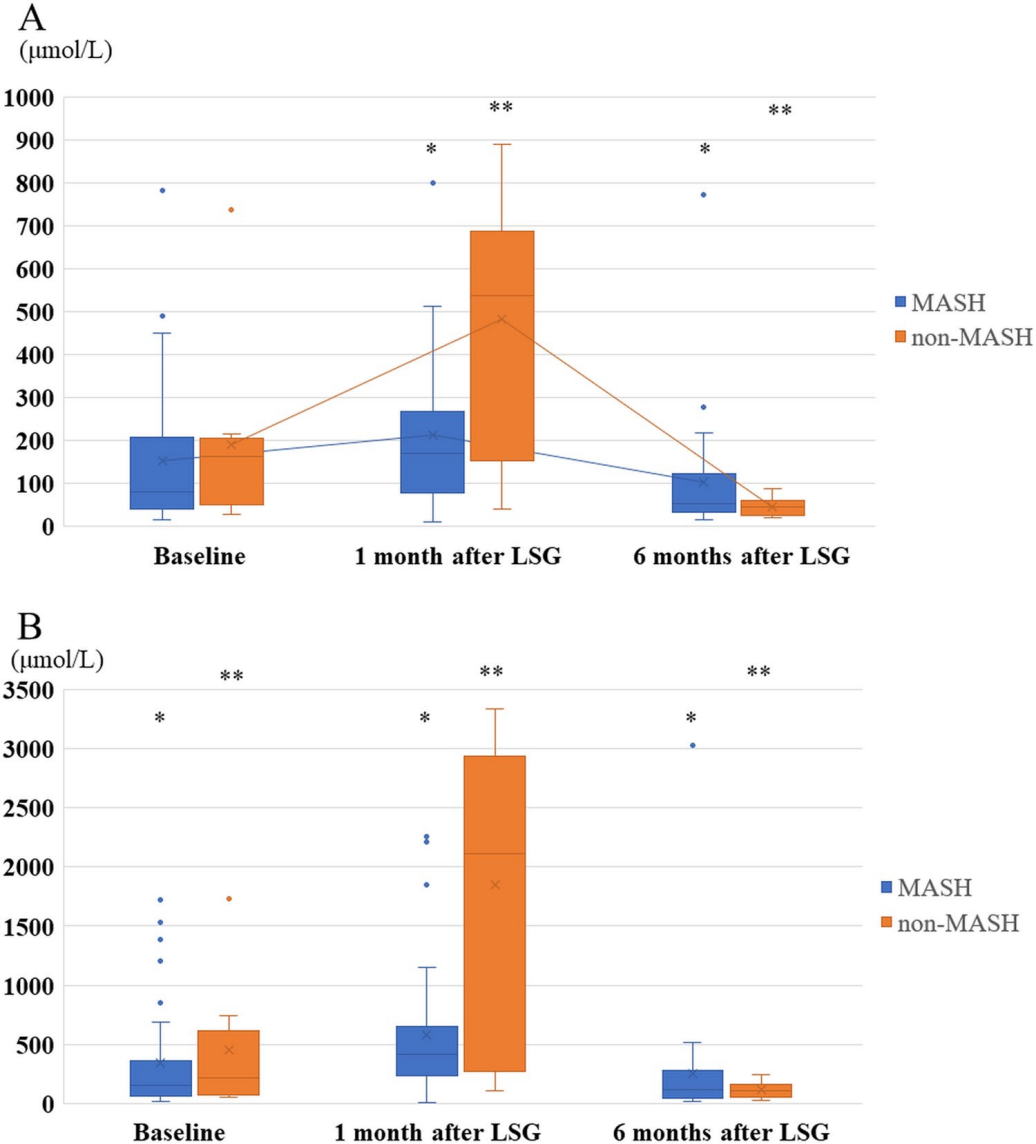
Comparison of AcAc and β-OHB levels between patients with T2D and without MASH. Both AcAc and β-OHB increased slightly after 1 month, decreased after 6 months, lower than the basal values in patients with MASH. ketone bodies in patients without MASH significantly increased at after 1 month after LSG, then both AcAc and β-OHB without MASH decreased at 6 months after LSG lower than in patients with MASH and own basal data. **A**: Changes of AcAc levels. * Serum AcAc levels in patients with MASH significantly decreased 6 months after LSG. (102.8 μmol/L vs. 211.3 μmol/L, *p* = 0.004). ** Serum AcAc levels in patients without MASH also significantly decreased at 6 months after LSG (45.3 μmol/L vs. 481.5 μmol/L, *p* = 0.003). **B**: Changes of β-OHB levels. * Serum β-OHB levels in patients with MASH significantly increased at 1 month after LSG (576.3 μmol/L vs. 347.0 μmol/L, *p* = 0.014), and then significantly decreased at 6 months after LSG (117.2 μmol/L vs. 576.3 μmol/L, *p* = 0.002). ** Serum β-OHB levels in patients without MASH (1845.6 μmol/L vs. 450.5 μmol/L,* p* < 0.001) at 1 month after LSG compared to the baseline, and then significantly decreased at 6 months after LSG (117.2 μmol/L vs. 1845.6 μmol/L,* p* < 0.001)

We also compared changes in ketone bodies between patients with/without MASH (Fig. 3). Both AcAc and β-OHB increased slightly after 1 month and decreased after 6 months lower than the basal values in patients with MASH. On the other hand, ketone bodies in patients without MASH significantly increased at after 1 month after LSG with several times of patients with MASH. However, Both AcAc and β-OHB without MASH decreased at 6 months after LSG lower than in patients with MASH and own basal data. At 1 month after LSG, AcAc was raised compared to the baseline; however, it was not significant (211.3 μmol/L vs. 151.3 μmol/L, *p* = 0.059) in patients with MASH. Conversely, AcAc levels in patients with MASH (102.8 μmol/L vs. 211.3 μmol/L, *p* = 0.004) and without MASH (45.3 μmol/L vs. 481.5 μmol/L, *p* = 0.003) significantly decreased 6 months after LSG. Concerning β-OHB changes, there were significant disparities in changes between patients with MASH (576.3 μmol/L vs. 347.0 μmol/L, *p* = 0.014) and without MASH (1845.6 μmol/L vs. 450.5 μmol/L, *p* < 0.001) 1 month after LSG compared to the baseline. Furthermore, in both groups, the β-OHB notably decreased 6 months after LSG. Regarding the comparison between the histopathological findings of intraoperative liver biopsy and ketone body levels 1 month after LSG, both AcAc (ρ = 0.775) and β-OHB (ρ = 0.834) were strongly correlated with liver steatosis. In addition, both AcAc (ρ = 0.750) and β-OHB (ρ = 0.803) were strongly correlated with NAFLD activity scores. In contrast, the pericellular fibrosis score was not correlated with dynamic changes in the ketone bodies after LSG.

The correlations between the levels of ketone bodies and the collected parameters at every measuring point are shown in Table 3. At baseline, both AcAc and β-OHB were positively correlated with body weight, BMI, HbA1c, AST, Type IV collagen 7S, and FIB-4 index. However, HDL-C levels were significantly lower in patients with high ketone body levels. At 1 month after LSG, TC, TG, LDL-C, HDL-C, and HS-CRP were negatively correlated with both AcAc and β-OHB. At 6 months after LSG, creatinine was positively correlated with both AcAc and β-OHB. Conversely, TC, TG, LDL-C, and eGFR were negatively correlated with both AcAc and β-OHB.

**Table 3:** Correlation analysis between ketone bodies and metabolic parameters at every measuring point

	Baseline				1 month after LSG				6 months after LSG			
Variables	AcAc (μmol/L)		β-OHB (μmol/L)		AcAc (μmol/L)		β-OHB (μmol/L)		AcAc (μmol/L)		β-OHB (μmol/L)	
	ρ	*p*-value	ρ	*p*-value	ρ	*p*-value	ρ	*p*-value	ρ	*p*-value	ρ	*p*-value
Body weight (kg)	0.354	0.021	0.389	0.010	− 0.041	0.784	− 0.142	0.344	0.036	0.832	− 0.005	0.975
BMI (kg/m^2^)	0.326	0.034	0.360	0.019	0.103	0.494	− 0.010	0.945	− 0.003	0.981	− 0.036	0.833
TWL (%)	−	−	−	−	0.173	0.247	0.109	0.470	0.204	0.238	0.172	0.321
SFA (cm^2^)	0.233	0.171	0.346	0.038	0.094	0.561	0.005	0.974	− 0.162	0.366	− 0.145	0.420
VFA (cm^2^)	− 0.153	0.370	− 0.177	0.301	− 0.228	0.156	− 0.297	0.062	0.041	0.818	− 0.032	0.857
LV (mL)	− 0.116	0.595	− 0.021	0.921	− 0.079	0.669	− 0.191	0.303	− 0.184	0.321	− 0.246	0.181
FG (mg/dL)	0.206	0.189	0.225	0.150	− 0.106	0.491	− 0.204	0.183	− 0.129	0.451	− 0.115	0.502
IRI (μIU/mL)	− 0.110	0.492	− 0.111	0.489	− 0.019	0.897	− 0.010	0.944	− 0.023	0.895	0.021	0.904
HbA1c (%)	0.338	0.030	0.360	0.020	− 0.065	0.671	− 0.144	0.342	− 0.119	0.486	− 0.111	0.519
HOMA − IR (no unit)	− 0.095	0.552	− 0.061	0.703	− 0.133	0.380	− 0.154	0.311	− 0.036	0.835	− 0.018	0.917
HOMA − β (no unit)	0.049	0.759	0.088	0.583	0.031	0.837	0.085	0.573	0.090	0.601	0.123	0.473
C − peptide (ng/mL)	0.018	0.910	0.039	0.804	− 0.328	0.062	− 0.307	0.081	0.009	0.959	− 0.029	0.874
AST (U/L)	0.405	0.007	0.458	0.002	0.036	0.810	− 0.029	0.845	− 0.114	0.505	− 0.124	0.470
ALT (U/L)	0.113	0.473	0.107	0.498	− 0.107	0.475	− 0.154	0.304	− 0.096	0.575	− 0.105	0.542
Ferritin (ng/mL)	0.322	0.037	0.284	0.067	− 0.108	0.483	− 0.112	0.465	− 0.159	0.361	− 0.100	0.567
Type IV collagen 7S (ng/mL)	0.359	0.019	0.368	0.016	− 0.112	0.467	− 0.123	0.425	− 0.092	0.604	− 0.178	0.313
Transferrin (ng/mL)	− 0.010	0.945	− 0.021	0.893	− 0.431	0.003	− 0.450	0.002	− 0.118	0.503	− 0.108	0.541
FIB − 4 index (no unit)	0.466	0.002	0.419	0.006	0.022	0.880	0.007	0.961	0.116	0.498	0.071	0.679
TC (mg/dL)	− 0.255	0.103	− 0.331	0.031	− 0.399	0.006	− 0.343	0.020	− 0.295	0.080	− 0.242	0.153
TG (mg/dL)	− 0.187	0.234	− 0.225	0.151	− 0.360	0.015	− 0.429	0.003	− 0.298	0.077	− 0.245	0.148
LDL − C (mg/dL)	− 0.149	0.344	− 0.220	0.160	− 0.340	0.021	− 0.287	0.055	− 0.309	0.065	− 0.278	0.100
HDL − C (mg/dL)	− 0.311	0.044	− 0.314	0.042	− 0.332	0.025	− 0.255	0.090	− 0.069	0.688	0.011	0.948
HS − CRP (mg/dL)	0.138	0.387	0.182	0.252	0.399	0.006	0.409	0.005	− 0.037	0.834	− 0.069	0.697
eGFR (mL/min/1.73m^2^)	0.013	0.931	0.069	0.662	0.135	0.368	0.097	0.519	− 0.391	0.018	− 0.459	0.048
BUN (mg/dL)	0.197	0.210	0.065	0.682	− 0.057	0.700	− 0.043	0.778	0.081	0.636	0.162	0.344
Creatinine (mg/dL)	0.156	0.322	0.106	0.502	− 0.237	0.116	− 0.215	0.155	0.347	0.037	0.401	0.015
Aldosterone (pg/mL)	0.033	0.862	0.041	0.832	− 0.030	0.914	0.006	0.981	− 0.305	0.249	− 0.268	0.314
U − Protein (mg/day)	− 0.057	0.719	− 0.067	0.676	0.172	0.262	0.149	0.333	0.160	0.381	0.197	0.279
U − Albumin (mg/L)	− 0.066	0.681	− 0.076	0.632	0.037	0.809	− 0.019	0.898	0.250	0.159	0.291	0.099

*LSG*, laparoscopic sleeve gastrectomy; *AcAc*, acetoacetic acid; *β-OHB*, β-hydroxybutyric acid; *BMI*, body mass index; *TWL*, total weight loss; *SFA*, subcutaneous fat area; *VFA*, visceral fat area; *LV*, liver volume; *FG*, fasting glucose; *IRI*, immunoreactive insulin; *HbA1c*, hemoglobin A1c; *HOMA-IR*, homeostasis model assessment-insulin resistance; *HOMA-β*, homeostatic model assessment beta cell function; *AST*, aspartate aminotransferase; *ALT*, alanine aminotransferase; *TC*, total cholesterol; *TG*, triglyceride; *LDL-C*, low-density lipoprotein cholesterol; *HDL-C*, high-density lipoprotein cholesterol; *HS-CRP*, high-sensitivity C-reactive protein; *eGFR*, estimated glomerular filtration rate; *BUN*, blood urea nitrogen; *U-protein*, urinary protein; *U-albumin*, urinary albumin

Moreover, we analyzed the relationships between changes in ketone bodies and weight loss and metabolic markers (Table 4). In the early phase, both Δ1AcAc and Δ1β-OHB mainly had strong positive correlations with changes in the T2D and MASLD parameters. In the middle term after LSG, both Δ2AcAc and Δ2β-OHB were positively correlated with changes in HS-CRP and U-protein.

**Table 4:** Correlation analysis between changes of ketone bodies and metabolic parameters

Variables	Δ1 = (value at 1 month after LSG) – (value at baseline)			
	AcAc (μmol/L)		β-OHB (μmol /L)	
	ρ	*p***-**value	ρ	*p***-**value
ΔVFA (cm^2^)	0.743	0.035		
ΔFG (mg/dL)	0.832	0.002	0.878	< 0.001
ΔHbA1c (%)	0.637	0.035	0.637	0.035
ΔHOMA-β (no unit)	− 0.813	0.026	− 0.826	0.022
ΔC-peptide (ng/mL)	0.844	0.009	0.900	0.002
ΔAST (U/L)	0.739	0.009	0.799	0.003
ΔALT (U/L)			0.640	0.034
ΔFerritin (ng/mL)	0.753	0.008	0.812	0.002
ΔType IV collagen 7S (ng/mL)	0.789	0.004	0.802	0.003
ΔFIB-4 index (no unit)	0.839	0.001	0.873	< 0.001
ΔBUN (mg/dL)	0.671	0.024	0.664	0.026
Variables	Δ2 = (value at 6 months after LSG) – (value at 1 month after LSG)			
	AcAc (μmol/L)		β-OHB (μmol /L)	
	ρ	*p*-value	ρ	*p*-value
ΔIRI (μIU/mL)	0.463	0.045	0.375	0.113
ΔHbA1c (%)	− 0.353	0.137	− 0.361	0.128
ΔHOMA-β (no unit)	− 0.423	0.090	− 0.477	0.052
ΔTG (mg/dL)	− 0.620	0.005	− 0.630	0.004
ΔLDL-C (mg/dL)	− 0.326	0.173	− 0.323	0.178
ΔHDL-C (mg/dL)	− 0.427	0.068	− 0.440	0.059
ΔHS-CRP (mg/dL)	0.496	0.030	0.547	0.015
ΔeGFR (no unit)	0.333	0.163	0.367	0.121
ΔCreatinine (no unit)	− 0.250	0.301	− 0.272	0.259
ΔU-protein (mg/day)	0.572	0.025	0.662	0.007

*LSG*, laparoscopic sleeve gastrectomy; *AcAc*, acetoacetic acid; *β-OHB*, β-hydroxybutyric acid; *VFA*, visceral fat area; *FG*, fasting glucose; *HbA1c*, hemoglobin A1c; *HOMA-β*, homeostatic model assessment beta cell function; *AST*, aspartate aminotransferase; *ALT*, alanine aminotransferase; *BUN*, blood urea nitrogen; *IRI*, immunoreactive insulin; *TG*, triglyceride; *LDL-C*, low-density lipoprotein cholesterol; *HDL-C*, high-density lipoprotein cholesterol; *HS-CRP*, high-sensitivity C-reactive protein; *eGFR*, estimated glomerular filtration rate; *U-protein*, urinary protein

## Discussion

In this study, we thoroughly analyzed changes in ketone bodies and the metabolic effects of LSG among Japanese patients with severe obesity. As previous articles demonstrated, serum ketone bodies reached peak values at 1 month after LSG [1, 14]. We first demonstrated that the preoperative ketone bodies in patients with T2D were significantly higher due to antidiabetic therapy; however, β-OHB in patients without T2D dramatically increased 1 month after LSG by prompt lipid metabolism. Furthermore, the changes in ketone bodies without MASH were similar to those of patients without T2D. We also first demonstrated that levels and changes in ketone bodies after LSG were mainly linked to lipid metabolism and the improvement of CKD. It was previously understood that ketone body surges after metabolic and bariatric surgery were evaluated as physiological phenomena because of glucose starvation and implemented increase of lipid metabolism. In this study, changes in serum ketone bodies after LSG were not simple physiological phenomena, and ketone body surges are playing important roles in adding higher therapeutic effects of LSG.

Ketone bodies constitute a family comprising three low-molecular-weight hydrosoluble molecules: acetone, AcAc, and β-OHB produced mainly by the liver when glucose is in short supply in the body. The productivity of ketone bodies depends on the mitochondrial expression of hydroxymethylglutaryl CoA synthase 2, the ketogenic rate-limiting enzyme leading to ketone body production using acetyl CoA and AcAc-CoA derived from the β-oxidation of excess fatty acids [15]. Ketogenesis and the use of ketone bodies as alternative fuels in specific organs are adaptive metabolic mechanisms when carbohydrates and glucose stores are limited [5]. This physiological mechanism works during the perioperative periods of the bariatric procedure. In addition, the administration of antidiabetic drugs, such as sodium-dependent glucose transporter 2 (SGLT-2) inhibitors and glucagon-like peptide-1 (GLP-1) receptor agonists, increases serum ketone bodies [1, 9]. These antidiabetic drugs have starvation effects by glucose excretion and dramatic hypoglycemic action; therefore, preoperative ketone bodies in patients with T2D were higher due to artificial glucose shortage. From these backgrounds, our results also displayed both phenomena using a stratified evaluation with/without T2D.

This study demonstrated that serum ketone bodies at 6 months after LSG were lower than basal values. Consumption of redundant accumulated fat gradually decreases because the weight-loss effect by LSG is higher at 6 months after LSG than baseline. However, both AcAc and β-OHB in patients with MASH at 6 months after LSG were higher than patients without MASH. We previously reported that weight-loss effects in patients with MASH were significantly lower than those without MASH like as T2D [16].

We first clarified that the early postoperative elevation of β-OHB in patients without T2D/MASH was markedly higher than in patients with/T2D/MASH. Moreover, higher liver steatosis and NAFLD activity scores correlated with early postoperative increases in ketone bodies. Ketogenesis is often used as a proxy for hepatic fat oxidation and impairments of ketogenesis emerge as MASLD progresses [17]. In the setting of ketogenic insufficiency in patients with severe obesity, lipogenesis and glucose production are increased due to overnutrition. However, metabolic and bariatric procedures can easily convert to ketogenic dominant conditions; therefore, fat metabolism also rapidly increases. These phenomena were induced by the massive consumption of excess fatty acids in patients without T2D/MASH because these patients have a more insulin-secreting function of pancreatic β-cells and early recovery of insulin sensitivity in various organs. We previously reported that circulating levels of liver fat-associated hepatokine, leukocyte cell-derived chemotaxin 2, were closely correlated with the severity of MASH, including steatosis and inflammation. Another hepatokine, selenoprotein P, raised insulin resistance and suppressed fat metabolism due to the role of the hunger hormone; therefore, lying MASH itself may be an important factor in suppressing lipid metabolism [18]. Nonetheless, patients with T2D/MASH might be categorized as having diabetic dyslipidemia (DD); DD is the concept of the complications of T2D and quantitative lipotoxicity to various organs due to strong insulin resistance [19]. In this study, patients without DD mainly received higher ketone body surges due to dramatic metabolic effects. Moreover, the early improvement of DD will help reduce cardiovascular risk by preventing endothelial damage.

Another emphasis of this study is the relationships between changes in ketone bodies and improved CKD. Recently, β-OHB has had renoprotective effects via various mechanisms, especially by administrating SGLT-2 inhibitors for patients with T2D [20, 21]. Ketone bodies reduce mitochondrial damage and oxidative stress by stimulating nuclear factor erythroid 2-related factor 2 translocation and activating the antioxidant pathway [22]. Furthermore, an increase in ketone bodies by SGLT-2 inhibitors causes tubulopathy and glomerular damage by suppressing the mammalian targets of rapamycin complex 1 signal activity [9]. Previous reports have found that the metabolic effects of CKD are gradually present in contrast to those for T2D [23]. Therefore, we could not find clear relationships between ketone bodies and CKD parameters in the early phase after LSG. Nevertheless, mid-term changes in ketone bodies had relationships with eGFR and albuminuria. This phenomenon may be caused by complicated mechanisms, such as improved hyperfiltration, enhanced inflammation, decreased renin–angiotensin–aldosterone system, and the antihypertensive effects of LSG in addition to ketone body activity [24]. To clarify the true correlations between ketone bodies and CKD after metabolic surgery, further detailed and powerful studies are warranted.

This study enrolled patients who underwent LSG to equalize therapeutic effects. On the other hand, patients who underwent other endorsed malabsorptive procedures were not enrolled in this study. Malabsorptive procedures naturally have better weight loss and metabolic effects on obesity-related diseases. Furthermore, probiotic supplementation and adjuvant administration of GLP-1 receptor agonists may have a potential for higher ketone body surges [25]. In the near future, further studies, including malabsorptive procedures and pre- and postoperative aggressive treatments, may clarify the role of ketone bodies in patients underwent metabolic and bariatric surgery.

Surgeons and allied healthcare professionals involved in metabolic and bariatric surgery want to know surrogate markers of postoperative therapeutic effects. Serum ketone bodies are currently easily and promptly measured in clinical practice; therefore, we will be able to evaluate the patient’s various metabolic conditions by evaluating changes in serum ketone bodies.

When interpreting the current study’s results, certain limitations must be considered. First, the number of patients we recruited was relatively small due to the requirement for written informed consent to enroll in this study. Second, the study involved only one institution and was retrospective; a single institutional observational study might produce selection bias and confounding bias. Third, all patients received only LSG; therefore, patients who underwent other bariatric procedures were not included in this study. Fourth, our results indicate only the early postoperative changes in ketone bodies, T2D, MASH, dyslipidemia, and CKD parameters. Fifth, there was selection bias due to previous limitations in this study. Therefore, well-designed prospective studies evaluating the long-term changes in ketone bodies after metabolic surgery may denote the further positions of ketone bodies as metabolism-improving factors.

## Conclusion

Our study first demonstrated that the postoperative surge of ketone bodies plays a crucial function in controlling metabolic effects after LSG. In patients without T2D, serum β-OHB was markedly higher than in patients with T2D at 1 month after LSG due to the prompt hypermetabolism of accumulated fat. Moreover, β-OHB in patients without MASH was significantly higher than that of patients with MASH. Mid-term changes in ketone bodies are closely correlated with CKD parameters. These findings suggest the cause and consequent roles of ketone bodies in the metabolic benefits of bariatric surgery.

## Data Availability

The datasets generated and/or analyzed during the current study are available from the corresponding author upon reasonable request. The data are not publicly available due to the duty of confidentiality of all patients set by the domestic law; therefore, all data have been anonymized and strictly protected from external network connection.

## Abbreviations

*LSG*: Laparoscopic sleeve gastrectomy
*BMI*: Body mass index
*T2D*: Type 2 diabetes
*TWL*: Total weight loss
*SFA*: Subcutaneous fat area
*VFA*: Visceral fat area
*LV*: Liver volume
*HbA1c*: Hemoglobin A1c
*AST*: Aspartate aminotransferase
*MASLD*: Metabolic dysfunction-associated steatotic liver disease
*TC*: Total cholesterol
*TG*: Triglyceride
*LDL-C*: Low-density lipoprotein cholesterol
*HDL-C*: High-density lipoprotein cholesterol
*HS-CRP*: High-sensitivity C-reactive protein
*eGFR*: Estimated glomerular filtration rate
*U-protein*: Urinary protein
*U-albumin*: Urinary albumin
*CKD*: Chronic kidney disease
*AcAc*: Acetoacetic acid
*β-OHB*: β-Hydroxybutyric acid
*CT*: Computed tomography
*HU*: Hounsfield unit
*MASH*: Metabolic dysfunction-associated steatohepatitis
*SGLT-2*: Sodium-dependent glucose transporter 2
*GLP-1*: Glucagon-like peptide-1
*DD*: Diabetic dyslipidemia
